# IL-23R mutation is associated with ulcerative colitis: A systemic review and meta-analysis

**DOI:** 10.18632/oncotarget.13607

**Published:** 2016-11-25

**Authors:** Ling-Long Peng, Ying Wang, Feng-Ling Zhu, Wang-Dong Xu, Xue-Lei Ji, Jing Ni

**Affiliations:** ^1^ Department of Science and Education, The Second People's Hospital of Wuhu, Wuhu, Anhui 241000, China; ^2^ Department of Environmental Health, Suzhou Municipal Center for Disease Prevention and Control, Suzhou, Jiangsu 215004, China; ^3^ Department of Rheumatology and Immunology, West China Hospital, Sichuan University, Chengdu, Sichuan 610041, China; ^4^ The Teaching Centre for Preventive Medicine, School of Public Health, Anhui Medical University, Hefei, Anhui 230032, China

**Keywords:** meta-analysis, ulcerative colitis, polymorphism, susceptibility, IL-23R

## Abstract

**Objectives:**

Since a genome-wide association study revealed that Interleukin-23 receptor (IL-23R) gene is a candidate gene for Ulcerative Colitis (UC), many studies have investigated the association between the IL-23R polymorphisms and UC. However, the results were controversial. The aim of the study was to determine whether the IL-23R polymorphisms confer susceptibility to UC.

**Methods:**

A systematic literature search was carried out to identify all potentially relevant studies. Pooled odds ratios (ORs) with 95% confidence intervals (CIs) were used to estimate the strength of association.

**Results:**

A total of 33 studies in 32 articles, including 10,527 UC cases and 15,142 healthy controls, were finally involved in the meta-analysis. Overall, a significant association was found between all UC cases and the rs11209026A allele (OR = 0.665, 95% CI = 0.604~0.733, *P* < 0.001). Similarly, meta-analyses of the rs7517847, rs1004819, rs10889677, rs2201841, rs11209032, rs1495965, rs1343151 and rs11465804 polymorphisms also indicated significant association with all UC (all *P* < 0.05). Stratification by ethnicity revealed that the rs11209026, rs7517847, rs10889677, rs2201841 andrs11465804 polymorphisms were associated with UC in the Caucasian group, but not in Asians, while the rs1004819 and rs11209032 polymorphisms were found to be related to UC for both Caucasian and Asian groups. However, subgroup analysis failed to unveil any association between the rs1495965 and rs1343151 polymorphisms and UC in Caucasians or Asians.

**Conclusions:**

The meta-analysis suggests significant association between IL-23R polymorphisms and UC, especially in Caucasians.

## INTRODUCTION

Ulcerative Colitis (UC) is a chronic relapsing and remitting intestinal inflammatory disorder that is confined to the mucosal and submucosal layers of rectum and colon, invading epithelial lining of the gut [1, 2]. The etiology and pathogenesis of UC are still unclear, but the epidemiological data have suggested that genetic variation is a major risk factor for the evolution of UC [3, 4]. Recently, a genome-wide association study (GWAS) in north Indian identified three novel human leucocyte antigen (HLA)-independent UC risk loci from chromosome 6 [5]. Furthermore, a novel locus at 6q22.1 was found to be associated with UC in a south European population [6]. To date, approximately 100 UC susceptibility loci have identified via GWAS studies and immnochip data, and these loci are implicated in the pathphysiologic mechanisms of UC, including microbe recognition, lymphocyte activation, cytokine signaling and intestinal epithelial defense [4–9]. Genetic studies provide clues on the immunopathogenesis for the disease, highlighting the role of immunity in the disease development of UC.

Interleukin-23 (IL-23) is a pro-inflammation cytokine secreted by activated macrophages and dendritic cells, which can influence the differentiation of native CD4+ T cells into T helper 17 subsets [10]. As a novel subset of Th cells, Th17 cells selectively secrete several pro-inflammatory cytokines, mainly IL-17, IL-6 and tumor necrosis factor (TNF-α) [11]. IL-23 binding to its receptor exerts profound effects through the Janus kinase (JAK)-signal transducer and activator of transcription (STAT) and NF-κB signaling pathways [12, 13]. Kenna *et al.* found that IL-23R was expressed at a significantly elevated level in the peripheral blood of ankylosing spondylitis (AS) patients, and the increased IL-23R levels were driven by large increases in the proportion of γ/δ T cells expressing IL-23R [14]. IL-17-secreting γ/δ T cells, responding rapidly to IL-23 signals, can enhance autoimmunity by restraining the function of regulatory T cells by an IL-23-dependent mechanism [15]. An IL-23R-mediated altered response to IL-23 signals in γ/δ cells can lead to overproduction of IL-17, suggesting a potential mechanism that increasing IL-23R expression might play a role in the human autoimmune disease [14]. IL-23/IL-23R signaling has been confirmed as a key conductor of innate and adaptive inflammatory responses in the intestinal mucosa [17]. Studies on mice models have indicated that IL-23/IL-23R is essential for intestinal inflammation of T cell-dependent colitis [11, 17]. Furthermore, researches on human UC showed increased IL-23R expression levels in colon tissues [18]. A genome-wide association study by Duerr *et al.* firstly revealed that IL-23R was a potential candidate gene for UC susceptibility [19]. IL-23R gene is mapped to the chromosome 1 (1p31.3) [12]. In the last decade, polymorphisms in this locus have been identified, and then, multiple studies focus on the association between these polymorphisms and UC [20–51]. However, the results of previous studies in different populations are inconsistent. This discrepancy may be attributed to the sample size differences, inadequate statistical power, various racial and ethnic backgrounds, etc.

Meta-analysis is a statistical method of augmenting the effective sample size through merging data from single association studies, thus enhancing the statistical power of the analysis for the estimation of genetic effects [52]. Liu *et al.* have performed a meta-analysis to assess the association between the IL-23R gene polymorphisms and UC risk, but this meta-analysis only included sixteen studies [53], and more studies concerning the association between single nucleotide polymorphisms (SNPs) and UC risk have not been included. Thus, it seems necessary to perform a comprehensive meta-analysis including the current published studies to investigate the relationships between the IL-23R gene polymorphisms and susceptibility to UC.

## RESULTS

### Study characteristics

According to the inclusion and exclusion criteria, 32 published articles were included in the meta-analysis. Of the 32 articles, one included two cohorts [20], thus, each cohort was regarded as a separate study. Finally, a total of 33 studies in 32 articles were identified to examine the association between the IL-23R gene polymorphisms and susceptibility to UC, involving 10,527 patients with UC and 15,142 healthy controls. Overall, five studies were performed among Asians, and the other studies among Caucasians. The main characteristics of the 32 studies were summarized in Table 1.

**Table 1:** Characteristics of the individual studies included in the meta-analysis

First author (Year)	Population (Ethnicity)	UC	Control	Genotyping	IL-23R polymorphisms studied	HWE (*P*-value)
Büning (2007)	Hungarian (C)	176	428	PCR	rs11209026	0.457
	German (C)	118	200	PCR	rs11209026	0.467
Cummings (2007)	UK Caucasian (C)	647	1149	Sequencing	rs1004819,rs7517847,rs10 489629,rs2201841,rs11209 026,rs1343151,rs11209032, rs1495965	NA
Glas (2007)	German (C)	456	1381	PCR	rs1004819,rs7517847,rs10 489629,rs2201841,rs11465804,rs11209 026,rs1343151,rs10889677,rs11209032, rs1495965	NA
Limbergen (2007)	British (C)	86	342	TaqMan	rs11209026	0.277
Oliver (2007)	Spanish (C)	222	342	TaqMan	rs1004819,rs7517847,rs104 89629,rs11209026,rs13431 51,rs10889677,rs11209032, rs1495965	0.346;0.124;0.023;0.001; 0.734;0.084;0.087;0.004
Roberts (2007)	New Zealander (C)	466	591	TaqMan	rs11209026	0.776
Tremelling (2007)	English people (C)	975	1345	Sequencing	rs1004819,rs10489629,rs11 465804,rs11209026,rs13431 51,rs10889677,rs11209032, rs1495965	NA
Lakatos (2008)	Hungarian (A)	149	149	PCR	rs11209026	NA
Lappalainen (2008)	Finnish (C)	459	292	Sequencing	rs1004819,rs10489629,rs22 01841,rs11465804,rs11209 026,rs1343151,rs10889677, rs11209032	NA
Latiano (2008)	Italian (C)	804	716	TaqMan	rs7517847,rs11209026	0.459;0.221
Lu (2008)	Chinese (A)	124	100	PCR	rs11209026	NA
Marquez (2008)	Spanish (C)	344	547	TaqMan	rs7517847,rs11209026	0.710;0.545
Okazaki (2008)	Canadian (C)	117	314	TaqMan	rs7517847,rs2201841,rs11209 026,rs10889677,rs1495965	0.910;0.591;0.725;0.455; 0.181
Venegas (2008)	Chilean (C)	62	59	PCR-RFLP	rs11209026	0.840
Weersma (2008)	Dutch (C)	207	893	Sequencing	rs11209026	0.828
Chen (2008)	Chinese (A)	40	50	PCR	rs11209026	NA
Weersma (2009)	Dutch-Belgian (C)	1442	1045	Sequencing	rs11209026	NA
Crotterill (2010)	British (C)	205	877	Sequencing	rs11209026	NA
Lacher (2010)	German (C)	132	253	PCR-RFLP	rs11209026,rs7517847	0.789;0.995
Mahurker (2010)	Indian (C)	411	442	Sequencing	rs11209026	NA
Mitrovic (2010)	Slovenian (C)	136	345	PCR-RFLP	rs7517847	0.056
Sventoraityte (2010)	Lithuanian (C)	123	186	TaqMan	rs11209026	0.002
Yang (2011)	Korean (A)	654	601	Sequencing	rs1004819,rs2201841,rs10889 677,rs11209032, rs1495965	0.378;0.434;0.367;0.891; 0.810
Zhao (2011)	Chinese (A)	135	134	Sequencing	rs1343151,rs11209032	0.791;0.668
Hayatbakhsh (2012)	Iranian (C)	85	100	PCR-RFLP	rs7517847,rs1004819	0.778;0.879
Safrany (2012)	Hungarian (C)	282	253	PCR-RFLP	rs7517847,rs2201841,rs10889 677,rs11209032	0.081;0.121;0.024;0.270
Kanaan (2012)	American (C)	276	435	PCR	rs11465804	NA
Yu (2012)	Chinese (A)	270	268	Sequencing	rs1004819,rs1495965,rs22 01841,rs7517847,rs104896 29,rs10889677,rs1343151, rs11209032	NA
Ebrahimi (2013)	Iranian (C)	67	78	PCR	rs1004819,rs2201841,rs10 889677,rs1495965,rs75178 47,rs10489629,rs11209026, rs1343151	0.650;0.821;0.821;0.324; 0.699;0.608;0.724;0.707
Mihaljevic (2013)	Croatian (C)	93	99	PCR	rs11209026	0.449
Skieceviciene (2013)	Lithuanian-Latvian (C)	444	1154	TaqMan	rs11209026,rs11465804,rs1004819,rs10889677	0.137;0.304;0.373;0.141
Sarlos (2014)	Hungarian (C)	320	316	PCR-RFLP	rs1004819,rs2201841	0.545

Abbreviations: UC: Ulcerative Colitis, C: Caucasian, A: Asian, NA: not available.

### Evaluation of heterogeneity and publication bias

**Figure 1:**
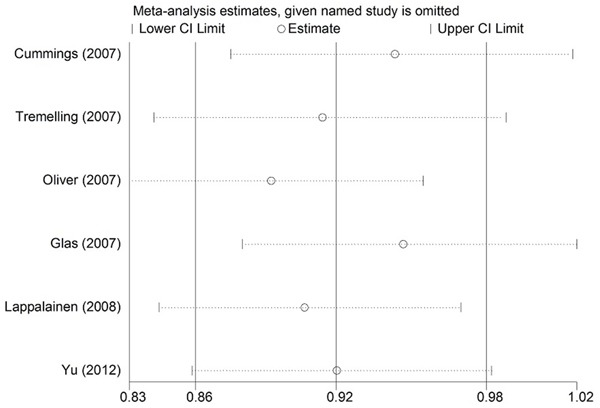
Sensitivity analysis of the summary odds ratio (OR) coefficients on the association between the IL-23R rs10489629 polymorphism and UC under allele contrast

**Figure 2:**
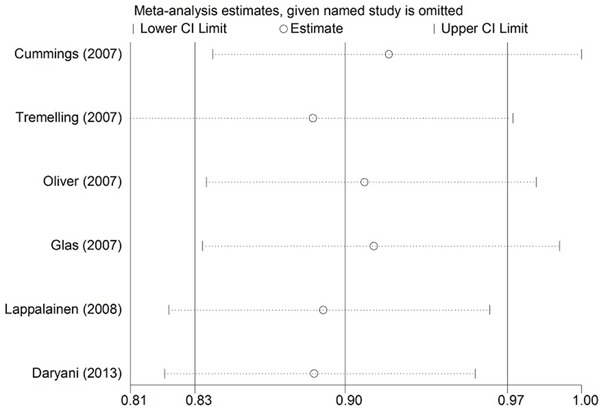
Sensitivity analysis of the summary odds ratio (OR) coefficients on the association between the IL-23R rs1343151 polymorphism and UC under allele contrast

Heterogeneity of the included studies regarding each polymorphism is presented in Table 2. Significant heterogeneity was found between the IL-23R rs10489629 polymorphism and UC in the overall and Caucasian populations (χ^2^ = 14.14, I^2^ = 64.9%, *P* = 0.015; χ^2^ = 14.14, I^2^ = 71.7%, *P* = 0.007, respectively). For the IL-23R rs1343151 polymorphism, stratification analysis by ethnicity detected significant heterogeneity in the Caucasian group (χ^2^ = 10.49, I^2^ = 52.3%, *P* = 0.063). Sensitivity analysis was adopted to assess the stability of the results and explore potential sources of heterogeneity across studies. We used leave-one-out method by sequentially omitting each study to assess the influence of individual data on the obtained results. (Figure 1, Figure 2). After omitting Oliver *et al.* [24] and Daryani *et al.* [48], respectively, no significant heterogeneity was found. Therefore, the two studies might be the potential sources of heterogeneity across studies.

**Table 2:** Meta-analysis of IL-23R polymorphisms in UC

Polymorphisms	Population	NO. of studies	Sample size		Test of association				Test of heterogeneity			Egger's test (*P*)
			Case	Control	OR (95% CI)	Z	*P*	Model	*χ*^2^	*P*	I^2^(%)	
rs11209026	Overall	25	8243	12899	0.665 (0.604~0.733)	8.25	<0.001	F	17.15	0.876	0.0	0.187
A versus G allele	Caucasian	23	8141	12799	0.662 (0.600~0.729)	8.30	<0.001	F	16.15	0.849	0.0	0.092
	Asian	2	102	100	1.004 (0.452~2.232)	0.01	0.992	F	0.03	0.864	0.0	NA
rs7517847	Overall	12	3589	5536	0.818 (0.768~0.871)	6.31	<0.001	F	5.78	0.887	0.0	0.535
G versus T allele	Caucasian	11	3319	5268	0.817 (0.766~0.871)	6.14	<0.001	F	5.76	0.835	0.0	0.570
	Asian	1	270	268	0.833 (0.652~1.063)	1.47	0.142	NA	NA	NA	NA	NA
rs1004819	Overall	11	4555	6994	1.196 (1.129~1.267)	6.09	<0.001	F	6.98	0.727	0.0	0.264
T versus C allele	Caucasian	9	3677	6131	1.208 (1.133~1.287)	5.80	<0.001	F	6.15	0.631	0.0	0.216
	Asian	2	878	863	1.146 (1.002~1.311)	1.98	0.047	F	0.35	0.553	0.0	NA
rs10889677	Overall	10	3948	5998	1.201 (1.127~1.280)	5.64	<0.001	F	7.24	0.612	0.0	0.461
A versus C allele	Caucasian	8	3025	5134	1.217 (1.134~1.306)	5.47	<0.001	F	5.99	0.541	0.0	0.539
	Asian	2	923	864	1.132 (0.976~1.313)	1.64	0.101	F	0.49	0.483	0.0	NA
rs2201841	Overall	9	3265	4638	1.163 (1.083~1.249)	4.15	<0.001	F	6.09	0.638	0.0	0.090
C versus T allele	Caucasian	7	2356	3783	1.175 (1.083~1.274)	3.87	<0.001	F	5.08	0.533	0.0	0.026
		7^a^	NA	NA	1.176 (1.084~1.275)	3.92	<0.001	F	NA	NA	NA	NA
	Asian	2	909	855	1.126 (0.971~1.307)	1.57	0.117	F	0.76	0.382	0.0	NA
rs11209032	Overall	9	4073	5752	1.127 (1.060~1.197)	3.85	<0.001	F	5.27	0.729	0.0	0.249
A versus G allele	Caucasian	6	3041	4762	1.116 (1.041~1.197)	3.10	0.002	F	4.82	0.438	0.0	0.044
		6^a^	NA	NA	1.120 (1.044~1.201)	3.12	0.002	F	NA	NA	NA	NA
	Asian	3	1032	990	1.160 (1.025~1.312)	2.35	0.019	F	0.16	0.922	0.0	0.312
rs1495965	Overall	8	3415	5473	1.073 (1.008~1.141)	2.22	0.026	F	7.19	0.409	2.6	0.392
G versus A allele	Caucasian	6	2492	4609	1.072 (0.999~1.149)	1.94	0.053	F	7.17	0.208	30.3	0.482
	Asian	2	923	864	1.076 (0.944~1.227)	1.09	0.274	F	0.02	0.892	0.0	NA
rs1343151	Overall	8	3241	4989	0.900 (0.836~0.969)	2.80	0.005	F	10.57	0.158	33.8	0.213
T versus C allele	Caucasian	6	2836	4587	0.916 (0.812~1.032)	1.45	0.148	R	10.49	0.063	52.3	0.142
	Asian	2	405	402	0.992 (0.513~1.922)	0.02	0.982	F	0.0	1.000	0.0	NA
rs10489629	Overall	6	3029	4777	0.944 (0.837~1.065)	0.93	0.350	R	14.14	0.015	64.9	0.259
G versus A allele	Caucasian	5	2759	4509	0.951 (0.829~1.090)	0.72	0.470	R	14.14	0.007	71.7	0.179
	Asian	1	270	268	0.914 (0.692~1.206)	0.64	0.525	NA	NA	NA	NA	NA
rs11465804	Overall	5	2530	4428	0.760 (0.639~0.904)	3.10	0.002	F	2.39	0.664	0.0	0.747
G versus T allele	Caucasian	5	2530	4428	0.760 (0.639~0.904)	3.10	0.002	F	2.39	0.664	0.0	0.747

Abbreviations: UC: Ulcerative Colitis, F: fixed effects model, R: random effects model, NA: not available.

* Adjusted using the “trim and fill” method.

**Figure 3:**
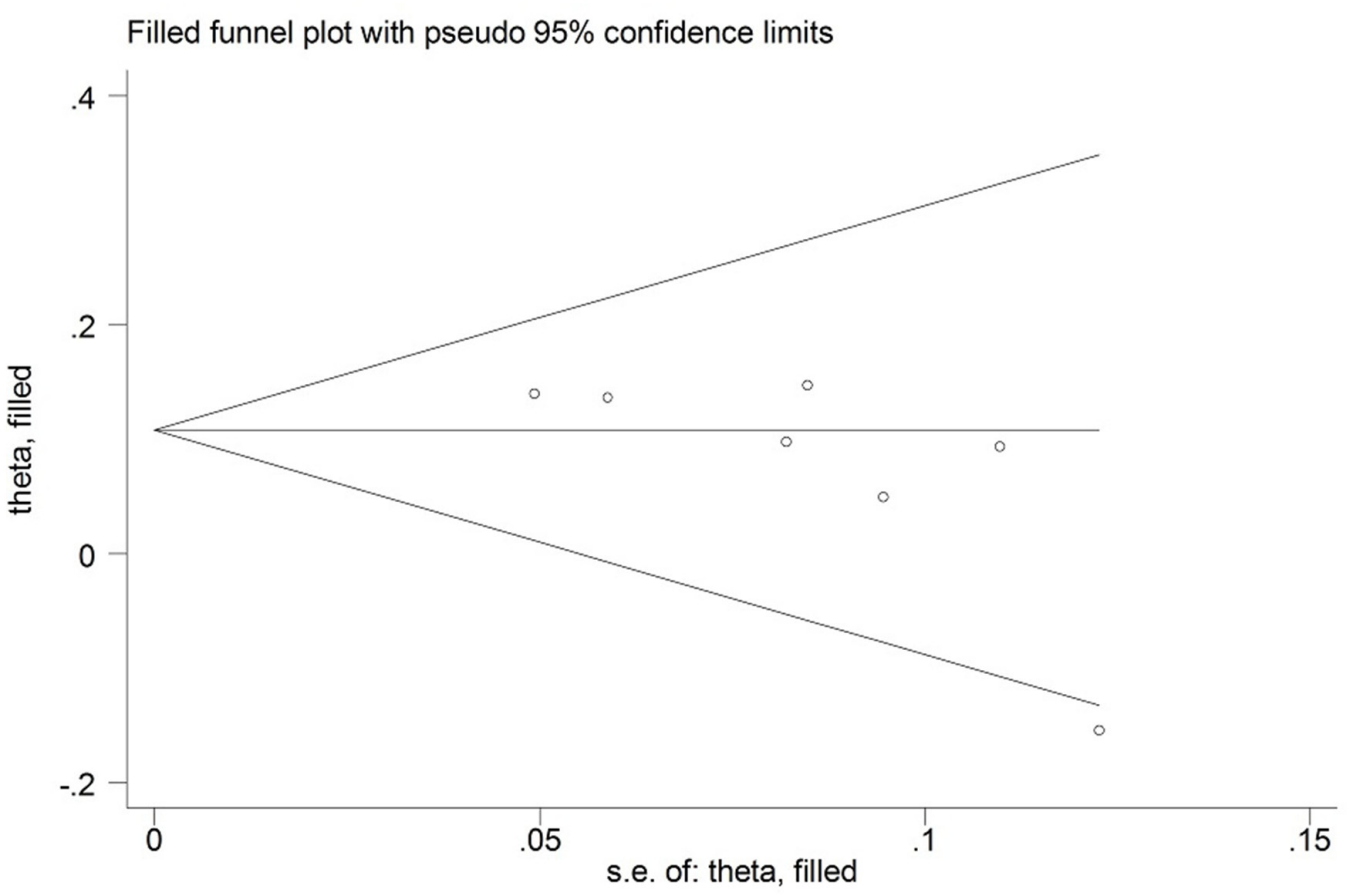
Funnel plots for meta-analysis of association between the IL-23R rs2201841 polymorphism and UC in the Caucasian population using the “trim and fill” technique

**Figure 4:**
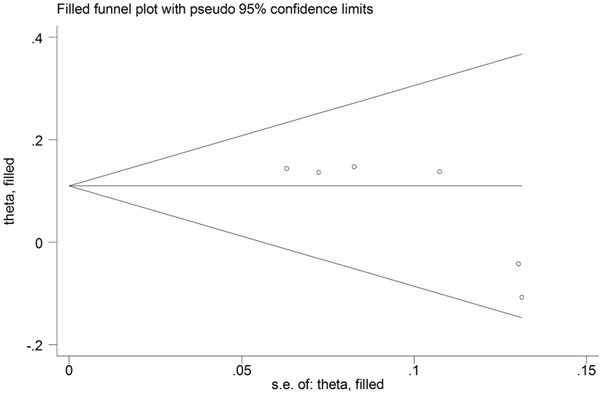
Funnel plots for meta-analysis of association between the IL-23R rs11209032 polymorphism and UC in the Caucasian population using the “trim and fill” technique

Evidence of publication bias was observed for the meta-analyses of the IL-23R rs2201841 and rs11209032 polymorphisms in the Caucasian population with a *P* value for Egger's linear regression test: 0.026 and 0.044. Then the “trim and fill” method was used to adjust for publication bias (Figure 3, Figure 4). Eventually, the adjusted OR calculation using the “trim and fill” technique remained significant (OR = 1.176, 95% CI = 1.084~1.275; OR = 1.120, 95% CI = 1.044~1.201, respectively). The results did not materially altered by publication bias, indicating the robust stability of the current conclusions.

### Meta-analysis of the IL-23R gene polymorphisms in Ulcerative Colitis

A summary of the meta-analysis of the association between the IL-23R gene polymorphisms and UC is presented in Table 2 and Supplementary Table S1.

### IL-23R rs11209026, rs7517847, rs10889677, rs2201841 polymorphisms and UC

**Figure 5:**
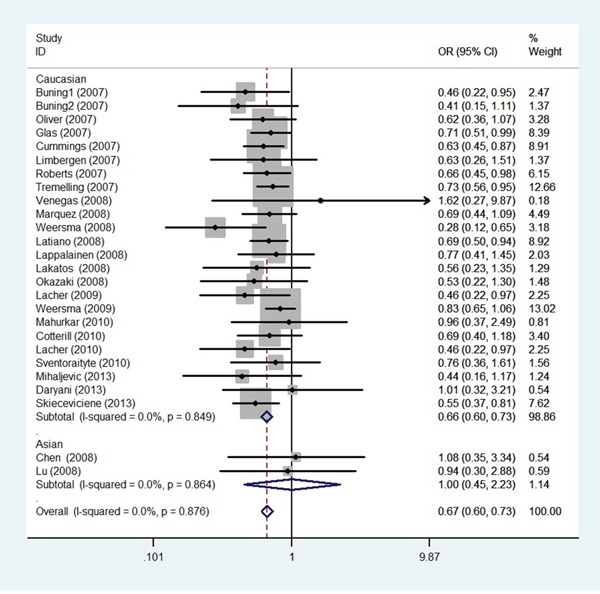
Odds ratios and 95% confidence intervals for individual studies and pooled data for the association between the A versus G allele of the IL-23R rs11209026 polymorphism and UC

Meta-analysis revealed that a significant association between the rs11209026A allele and UC risk in the overall population (OR = 0.665, 95% CI = 0.604~0.733, *P* < 0.001; Figure 5). Furthermore, stratification by ethnicity indicated that the rs11209026A allele was significantly associated with UC risk in the Caucasian population (OR = 0.662, 95% CI = 0.600~0.729, *P* < 0.001; Figure 5), but not in the Asian population. Meta-analyses of the rs7517847, rs10889677, rs2201841 polymorphisms also showed the same pattern as for the results of rs11209026. All these polymorphisms were found to be related to UC susceptibility in Caucasians, but not in Asians (Table 2).

### IL-23R rs1004819, rs11209032 polymorphisms and UC

Overall, meta-analysis of the IL-23R rs1004819 and rs11209032 polymorphisms showed significant association between UC and the minor alleles in all study subjects (OR = 1.196, 95% CI = 1.129~1.267, *P* < 0.001; OR = 1.127, 95% CI =1.060~1.197, *P* < 0.001) (Table 2). Meanwhile, significant associations were also detected between these two polymorphisms and UC susceptibility in both Caucasians and Asians through the subgroup analysis stratified by ethnicity.

### IL-23R rs1495965, rs1343151 polymorphisms and UC

Meta-analysis showed significant association between the minor alleles of the rs1495965 and rs1343151 polymorphisms and the risk of UC in the overall population (OR = 1.073, 95% CI = 1.008~1.141, *P* < 0.001; OR = 0.900, 95% CI = 0.836~0.969, *P* = 0.005, respectively). However, ethnicity-specific analysis failed to identify any associations between the two polymorphisms and UC risk in neither Caucasian nor Asian subjects.

### IL-23R rs10489629, rs11465804 polymorphism and UC

No association was found between UC and the IL-23R rs10489629 polymorphism by meta-analyses (Table 2). For the IL-23R rs11465084 polymorphism, a significant association was observed in the G versus T allele in the Caucasian origin (OR = 0.760, 95% CI = 0.639~0.904, *P* = 0.002). However, there is no such study performed in the Asian group.

## DISCUSSION

UC is characterized by chronic intestinal inflammation as a result of an aberrant immune response. Considerable progress has been made in understanding the pathogenesis of UC. An inappropriate muscosal immune response to commensal bacterial flora induced by the interaction of environmental and genetic factors has been proposed [54]. Amounts of genetic association studies have identified many genes including nucleotide oligomerization domain 2 (NOD2), autophagy genes, STAT3, Cytotoxic T lymphocyte associated antigen-4 (CTLA-4) and genes whose products function in the IL-23/Th17 pathway, which play an important role in perpetuating the abnormal inflammatory response, have been considered as candidate genes in disease initiation of UC [3, 29, 32]. Genetic polymorphisms of the IL-23R gene have been investigated in many autoimmune diseases, and researches have showed that IL-23R gene is associated with the etiology of AS, psoriasis, rheumatoid arthritis (RA), and multiple sclerosis, inflammation bowel disease (IBD) [19, 55]. Since a genome wide association study by Duerr *et al.* firstly showed strong correlation of the IL-23R polymorphisms and UC, numerous studies have been carried out to detect the association between IL-23R polymorphisms and UC [19]. However, different research teams obtained contradictory results in different populations. For example, a study from Hayatbakhsh *et al.* indicated no association between the IL-23R gene polymorphisms rs7517847 and rs1004819 and the susceptibility of UC in an Iranian population [17]. Similarly, a study in a Finnish population demonstrated that the IL-23R polymorphisms (rs1004819, rs10489629, rs2201841, rs11465804, rs11209026, rs1343151, rs10889677, rs11209032) were not related to UC [28], while a study in England and Scotland revealed an association between the IL-23R polymorphisms (rs1004819, rs11209026, rs10889677, rs11209032) and UC [26]. Therefore, to better comprehend the relationship of the IL-23R gene polymorphisms and UC, we summarize the inconsistent results by meta-analysis to overcome small sample size problems of individual studies and enhance the statistical power and draw a more comprehensive and reliable conclusion.

**Figure 6:**
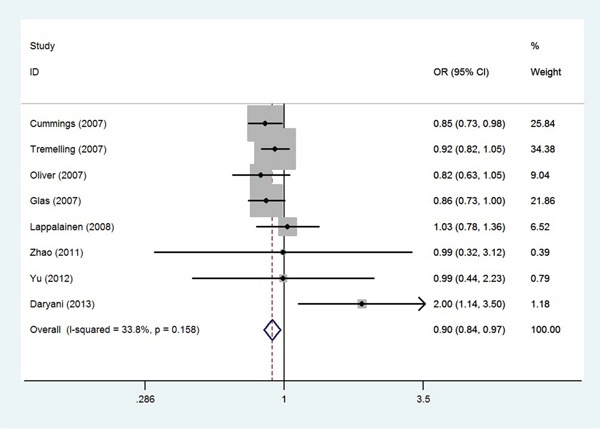
Odds ratios and 95% confidence intervals for individual studies and pooled data for the association between the T versus C allele of the IL-23R rs1343151 polymorphism and UC

The meta-analysis was conducted to discuss the association of ten polymorphisms in the IL-23R gene (rs11209026, rs7517847, rs1004819, rs10889677, rs2201841, rs11209032, rs1495965, rs1343151, rs10489629, rs11465804) and UC. Overall, IL-23R gene polymorphisms (except for rs10489629) are associated with UC risk. We also undertook subgroup analyses by ethnicity for these polymorphisms. Our findings provide strong evidence of a significant association between the IL-23R polymorphisms, with the exception of the rs1495965 and rs1343151 polymorphisms, and UC in Caucasians. The present study showed that the IL-23R rs1495965 and rs1343151 polymorphisms were not associated with UC in Caucasians and Asians. The reasons for this disagreement might arise from the followings. We found that the ORs (95% CIs) of the individual studies [22, 24, 26] included in meta-analysis of rs1343151 polymorphism were approximates to critical values (shown in Figure 6). If these studies increased the sample size, they might yield significant association. Similarly, ethnicity-specific analysis found that the IL-23R rs1004819 and rs11209032 polymorphisms were associated with UC susceptibility in Caucasians and Asians. However, polymorphisms studied, such as the rs11209026, rs7517847, rs10889677 and rs2201841 polymorphisms, revealed a different association with UC between Caucasians and Asians.

The diverse roles of the same gene polymorphism in subgroup analysis by ethnicity could be ascribed to the followings. It is well known that UC is the most prevalent in North America and Europe, and the least prevalent among African Americans and Asians [56]. There is a susceptibility factor for UC in Caucasians, but not in Asians. It suggested that IL-23R gene might have a susceptibility nature in the Caucasians, while it was not suggested to have such a nature in the Asians. In addition, the two polymorphisms were found to confer susceptibility to both the populations, but the number of individual studies included and sample size of pooled data in Asians in the current meta-analysis were small, increasing the sample size might change the results, so the conclusions drawn from this limited number of study subjects might not look necessarily reliable.

With regard to the functional effects of IL-23R polymorphisms, rs10889677, a variant located in the 3′-untranslated region of the IL-23R gene, have been demonstrated to induce the loss of microRNA regulation and enhance the IL-23R mRNA levels and protein production. In combination with this variant (rs10889677), miRNA-mediated dysregulation of IL-23 signaling strongly associated with UC susceptibility [57]. Furthermore, rs10889677 SNP also can change the function of its receptor by promoting its overexpression, skewing T cells to differentiate towards Th17 leading to inflammation by increasing release of other cytokines [58]. Another intronic polymorphism Arg381Gln (rs11209026), located in 3′-region of exon 9 of IL-23R, is significantly associated with UC susceptibility. Yu *et al.* found the protective allele A of rs11209026 SNP could increase the expression of the soluble form of IL-23R mRNA and diminish the induction of Th17 cells upon IL-23 stimulation [59].

The strength of our meta-analysis could be summarized as the followings. We did our best to identify as many published articles as we could through various searching approaches. Compared with the previous meta-analysis [53], the current study involved a total of 32 articles, which is much larger than the data from the previous meta-analysis. Thus, results of current study are more reliable, showing that IL-23R might be a potential therapeutic target for UC in combination with findings of GWAS studies and immnochip data. In addition, Jurgens *et al.* has examined the association of the IL-23R gene polymorphisms as a genetic predictor of infliximab responsiveness in UC patients and showed that UC patients carrying the IBD risk-increasing IL-23R polymorphisms were more likely to respond to infliximab than patients carrying the IBD risk-decreasing IL-23R polymorphisms (74.1% vs. 34.6%, *P* = 0.001), which suggests that IL-23R gene is related to treatment of UC patient [60]. Therefore, further research, involving larger and more diverse UC populations, are necessary in future, which in turn could lead to more accurate diagnosis and ultimately to the development of better drugs and more effective therapeutic strategies. Moreover, we performed subgroup analyses by ethnicity to look at the ethnic effect on the risk of UC. Besides, the “trim and fill” method was used to adjust for publication bias.

Certainly, some limitations of the present study should be addressed. First, this study could not analyze the potential gene-environment interactions and gene susceptibility haplotypes due to insufficient data. Second, our literature search was only limited on English and Chinese, language bias might exist. Third, significant between-study heterogeneity and publication bias was found in some analyses, which might have an influence on the current study. Fourth, some results are still controversial owing to relatively small sample size in Asians, and the results of meta-analysis were applicable only to the Asian and Caucasian groups.

In summary, the current study provides a comprehensive examination of the available evidence for the association between polymorphisms in the IL-23R gene and UC. The meta-analysis suggests that IL-23R gene polymorphisms are associated with UC susceptibility, especially in Caucasians. However, larger sample size studies taking environmental risk factor into account and including other ethnic groups are need to confirm the results from our meta-analysis in the future.

## MATERIALS AND METHODS

### Publication research

A systematic literature search in PubMed database, Elsevier Science Direct, China National Knowledge Infrastructure database (CNKI), Wanfang Database, and Chinese Biomedical database (CBM) was carried out to identify potential articles and references in the identified articles were checked manually to find additional studies concerning the association between polymorphisms in the IL-23R gene and UC risk. The text words were as follows: “ulcerative colitis or inflammatory bowel disease or UC or IBD” and “Interleukin-23 receptor or IL-23R or IL23R” combined with “gene or polymorphism or variant”. The languages were limited to English and Chinese. The last search was updated on May 1, 2016.

### Inclusion and exclusion criteria

A study was included in the analysis if a) it was a case-control or cohort study; b) it included original data (independence among studies); c) the studies tested the association between the IL-23R gene polymorphisms (rs11209026, rs7517847, rs1004819, rs10889677, rs2201841, rs11209032, rs1495965, rs1343151, rs10489629, rs11465804) and UC risk, and d) the studies provided sufficient data to calculate the statistical indicators. Studies were excluded if one of the followings existed: a) studies contained overlapping data; b) studies in which family members had been studied because of the analysis based on linkage considerations.

### Data extraction

The following data were extracted independently by two authors from each eligible article: first author's last name, publication year, population, ethnicity, numbers of cases and controls, genotyping methods, polymorphisms in the IL-23R gene studied and Hardy-Weinberg equilibrium (HWE) *P*-value.

### Statistical analysis

Allele frequencies at the IL-23R gene polymorphisms from each single study were determined by the counting method. The pooled odds ratios (ORs) and 95% confidence intervals (CIs) were calculated to assess the strength of association between these gene polymorphisms and UC susceptibility. HWE was examined using the χ^2^ test.

The χ^2^-test based on the Q statistic was applied to examine the heterogeneity of between-study [61]. If *I^2^* was more than 50% and *P* value was less than 0.1, the heterogeneity was considered to be significant, and then the random effects model was selected for meta-analysis. Otherwise, a fixed effects model was adopted.

Publication bias was estimated by Egger's linear regression test and the Funnel plot. If the *P* value was less than 0.05, statistically significant publication bias might exist [62].

All the statistical analyses of meta-analysis were performed by STATA statistical software (version 11.0 STATA Corp LP, College Station, TX, USA). A two-sided *P* value < 0.05 was regarded as statistically significant.

## SUPPLEMENTARY TABLE



## Abbreviations

GWAS: genome-wide association study
HLA: human leucocyte antigen
TNF-α: tumor necrosis factor
SNP: single nucleotide polymorphism
UC: ulcerative colitis
IL-23R: interleukin-23 receptor
JAK: Janus kinase
STAT: signal transducer and activator of transcription
AS: ankylosing spondylitis
RA: rheumatoid arthritis
IBD: inflammation bowel disease
CTLA-4: Cytotoxic T lymphocyte associated antigen-4
HWE: Hardy-Weinberg Equilibrium
OR: odds ratio
CI: confidence interval
CNKI: China National Knowledge Infrastructure database
CBM: Chinese Biomedical database
