# Supplementary material for: IL-23R mutation is associated with ulcerative colitis: A systemic review and meta-analysis
Source: Oncotarget. 2016 Nov 25;8(3):4849–63. doi: 10.18632/oncotarget.13607 (PMC5354875; doi:10.18632/oncotarget.13607)
Supplement: Supplementary file 1 [file oncotarget-08-4849-s001.pdf]

IL-23R mutation is associated with ulcerative colitis: A systemic review and meta-analysis

SUPPLEMENTARY TABLE

Supplementary Table S1: Meta-analysis of the IL-23R polymorphisms in UC under homozygote contrast, dominant model and recessive model

Polymorphisms	NO. of studies	Sample size		Test of association					Test of heterogeneity		Egger's test (*P*)
		Case	Control	OR(95% CI)	Z	*P*	Model	χ^2	*P*	I^2(%)	
rs11209026 AA vs AG+GG	10	2943	5180	0.352(0.145~0.855)	2.31	0.021	F	2.04	0.991	0.0	0.777
AA+AG vs GG	15	3385	5957	0.624(0.531~0.735)	5.68	<0.001	F	14.23	0.433	1.6	0.360
AA vs GG	10	2943	5180	0.339(0.139~0.823)	2.39	0.017	F	1.98	0.992	0.0	0.765
rs7517847 GG vs GT+TT	9	2216	2947	0.788(0.672~0.924)	2.94	0.003	F	6.44	0.598	0.0	0.284
GG+GT vs TT	9	2216	2947	0.793(0.706~0.891)	3.92	<0.001	F	9.53	0.299	16.1	0.424
GG vs TT	9	2216	2947	0.690(0.580~0.821)	4.18	<0.001	F	3.61	0.890	0.0	0.370
rs1004819 TT vs TC+CC	6	1748	2559	1.135(0.956~1.347)	1.45	0.148	F	0.92	0.969	0.0	0.358
TT+TC vs CC	6	1748	2559	1.263(1.105~1.444)	3.43	0.001	F	8.29	0.141	39.7	0.366
TT vs CC	6	1748	2559	1.225(0.999~1.501)	1.95	0.051	F	3.26	0.660	0.0	0.492
rs10889677 AA vs AC+CC	6	1788	2712	1.219(1.036~1.436)	2.38	0.017	F	2.40	0.792	0.0	0.195
AA+AC vs CC	6	1788	2712	1.252(1.011~1.550)	2.06	0.039	R	9.67	0.085	48.3	0.800
AA vs CC	6	1788	2712	1.300(1.041~1.621)	2.32	0.020	F	4.75	0.448	0.0	0.172
rs2201841 CC vs CT+TT	5	1433	1548	1.172(0.973~1.413)	1.67	0.095	F	2.83	0.587	0.0	0.948
CC+CT vs TT	5	1433	1548	1.078(0.904~1.287)	0.84	0.402	F	3.93	0.415	0.0	0.084
CC vs TT	5	1433	1548	1.132(0.860~1.491)	0.88	0.377	F	5.01	0.286	20.2	0.881
rs11209032 AA vs AG+GG	4	1266	1317	1.150(0.937~1.411)	1.34	0.181	F	4.42	0.220	32.1	0.392
AA+AG vs GG	4	1266	1317	1.027(0.871~1.211)	0.32	0.750	F	1.91	0.592	0.0	0.988
AA vs GG	4	1266	1317	1.130(0.894~1.428)	1.02	0.308	F	3.62	0.306	17.0	0.455
rs1495965 GG vs GA+AA	4	1067	1330	0.983(0.816~1.186)	0.17	0.862	F	5.89	0.117	49.0	0.949
GG+GA vs AA	4	1067	1330	1.030(0.853~1.245)	0.31	0.757	F	0.76	0.860	0.0	0.322
GG vs AA	4	1067	1330	1.042(0.826~1.313)	0.35	0.729	F	2.27	0.518	0.0	0.770
rs1343151 TT vs TC+CC	3	434	554	0.629(0.379~1.046)	1.79	0.074	F	0.26	0.611	0.0	0.317
TT+TC vs CC	3	434	554	0.750(0.559~1.006)	1.92	0.055	F	2.07	0.354	3.6	0.832
TT vs CC	3	434	554	0.577(0.338~0.985)	2.02	0.044	F	0.48	0.488	0.0	0.730

Abbreviations: UC: Ulcerative Colitis, F: fixed effects model, R: random effects model.
